# Effect of health belief model-based educational intervention on prostate cancer prevention; knowledge, practices, and intentions

**DOI:** 10.1186/s12885-024-12044-9

**Published:** 2024-03-04

**Authors:** Marwa Ibrahim Mahfouz Khalil, Ayat Ashour, Reem Said Shaala, Rasha Mahmoud Allam, Thoraya Mohamed Abdelaziz, Enas Fouad Sayed Mousa

**Affiliations:** 1 Gerontological Nursing, Faculty of Nursing, Alexandria University, Alexandria, Egypt; 2 Family Health Department, High Institute of Public Health, Alexandria University, 165 El Horeya Avenue, 21561 Alexandria, Egypt; 3 Internal Medicine, Geriatric Unit, Faculty of Medicine, Alexandria University, Alexandria, Egypt; 4 Cancer Epidemiology and Biostatistics, National Cancer Institute, Cairo University, Cairo, Egypt; 5 Medical-Surgical Nursing, Faculty of Nursing, Alexandria University, Alexandria, Egypt; 6 Geriatric Medicine and Gerontology, Faculty of Medicine, Helwan University, Cairo, Egypt

**Keywords:** Health belief model, Knowledge, Prostate cancer, Screening, Practices, Intention

## Abstract

**Background:**

Prostate cancer screening is a crucial preventive element for improving the survival rates of prostate cancer. Therefore, our research objective was to investigate the effect of health belief model-based education on prostate cancer knowledge, health beliefs, and preventive health practices among adult and older adult males.

**Methods:**

A one-group pre-test/post-test quasi-experimental study design was carried out at the one-day outpatient clinics affiliated to General Alexandria Main University Hospital. We enrolled 110 men aged 45–75 years old in a health belief model-based educational intervention program. Various questionnaires were utilized to gather data before, immediately after, and three months following the intervention. These questionnaires included the socio-demographic questionnaire, Prostate Cancer Knowledge Questionnaire (PCKQ), Prostate Cancer Screening-Health Belief Model Scale (HBM-PCS), Prostate Cancer Preventive Practices Questionnaire (PCPPQ), and one question regarding the intention to undergo PC screening.

**Results:**

Participants’ knowledge about prostate cancer screening improved significantly immediately after the program and this positive change was maintained at the follow-up (*p* = 0.000). Furthermore, participants’ perceptions and preventive practices towards prostate cancer screening had changed significantly after program completion and at follow-up (*p* = 0.000). After program completion, many of the participants (92.7%) expressed their intention to undergo prostate cancer screening within the coming six months (*p* = 0.000). The younger age group (45–49 years) showed higher scores in their perception of prostate screening (*p* = 0.001). Higher education and income were significantly associated with higher scores in the three scales (*p* = 0.000 in all scales).

**Conclusion:**

The study findings emphasized the effectiveness of the designed health educational program based on the HBM on PC preventive behaviors, through significantly improving participants’ knowledge level, perceptions, practices, and intentions to PC screening. The program is highly recommended for prostate cancer preventive health practices among both adult and older adult males.

**Supplementary Information:**

The online version contains supplementary material available at 10.1186/s12885-024-12044-9.

## Introduction

Prostate cancer (PC) is a substantial public health concern for men globally, ranking among the top five cancers that threaten their well-being. It affects approximately 1.1 million worldwide, [1] and accounted for 14.1% of all diagnosed cases with cancer in 2020 [2] It is the most commonly diagnosed cancer in men in more than half (112 of 185) of the countries, with mortality rates being particularly high in 48 countries based on GLOBOCAN 2020 Estimates [3]. 

In Africa, age-standardized incidence and mortality rates for prostate cancer stand at 26.6 and 14.6 per 100,000 men, respectively [4]. Egypt recorded a prostate cancer death rate of 0.30% of total deaths as per the WHO’s 2018 data, with an age-adjusted death rate of 7.72 per 100,000 population, ranking it 153rd globally [5, 6]. Though fewer than 30% of all incidences of PC are from developing countries, these countries have previously been estimated to have the highest mortality from PC due to late diagnosis [7]. Survival rates for prostate cancer patients are significantly higher when the disease is detected in its early stages. In fact, research shows that patients diagnosed with the earliest stage of prostate cancer have a 100% five-year survival rate, compared to less than 33% for those diagnosed at a later stage. Given these statistics, it’s essential to prioritize knowledge assessment and early diagnosis of prostate cancer to improve outcomes [8]. 

The risks of PC highlight the importance of preventive practices, as lack of awareness, preventive strategies, negative beliefs, and increased life expectancies contribute to 57% of all new cancer cases around the world [9]. Identifiable barriers to early detection and screening of PC include financial issues, lack of health insurance, poor health-seeking behavior, and a lack of cultural familiarity, training, or resources. These barriers, combined with a fear of cancer screening procedures and a lack of familiarity with health prevention, are significant impediments to the utilization of health and preventive services [10, 11]. In Egypt, the male population has limited awareness and knowledge of the disease and voluntary screening [12]. Additionally, men’s fatalistic beliefs and misconceptions concerning prostate cancer contribute to late reporting to healthcare settings and delay in seeking medical attention [13]. 

Primary prevention is a highly effective strategy that holds promise for long-term benefits for individuals diagnosed with prostate carcinoma. It is heartening to note that up to 50% of all cancers can be prevented [14]. In accordance with the American Cancer Society guidelines, men with an average risk profile should consider informed and shared decision-making with their healthcare provider regarding PSA testing beginning at the age of fifty years. For men with a higher risk profile, consideration should commence at the age of forty-five years. This approach enables individuals to make informed choices by weighing the potential benefits, risks, and uncertainties associated with the screening process [15]. 

Egypt has made significant strides in the fight against cancer, an achievement that is highly commendable. Since 2018, the country has implemented the Egypt National Multisectoral Action Plan for Prevention and Control of Noncommunicable Diseases, with the primary objective of reducing premature mortality rates by 15%. This plan is centered on mitigating risk factors, enhancing early detection, and ensuring effective treatment. To achieve this objective, a national cancer committee has been established to develop and implement a comprehensive national plan and guidelines for cancer control and early detection, with a particular emphasis on prostate cancer [16]. These efforts are in line with the Sustainable Development Goal 3.4, which seeks to reduce premature mortality from non-communicable diseases by one-third through prevention and treatment by 2030 [17]. 

The Health Belief Model (HBM) is a widely employed and well-established framework for the analysis of healthy behaviors. Originally devised in the 1950s by a group of social psychologists under the employ of the United States Public Health Service, the model aims to elucidate the means by which health educators can promote preventive behaviors and health screenings. The HBM revolves around the attitudes and beliefs of individuals with regard to cancer screening and prognosticates the likelihood of an individual taking action based on their perceptions of potential illness, the consequences of illness, and the perceived benefits and barriers associated with participation in the behavior [18]. Furthermore, the HBM integrates health motivation as a forecast of health-related behaviors, which encompasses a generalized state of intent that leads to behaviors aimed at preserving or enhancing health [19]. 

It is not known to what extent exposure to cancer screening information based on the health belief model influences clients’ decisions to participate in cancer screening. Little is researched about the age-related differences in the realm of prostate cancer prevention and related perceived screening behavior. One formative research among African Americans revealed that both younger/middle-aged and older men were considered the hardest to reach for prostate cancer education programs [20]. 

For that, the current research aimed at exploring this issue and shed light on determining the effect of health belief model-based teaching (perceived susceptibility, seriousness, motivation, barriers, and benefits) on preventive health practices regarding prostate cancer among males. This would help in providing a specifically tailored health education based on the model regarding screening and early identification and treatment of such ignored practices.

The study holds significant importance as it aims to fill a substantial gap in the existing literature concerning prostate cancer screening and preventive behaviors particularly crucial for men within the context of a reserved Arabic and Islamic culture or those who may have limited awareness of the healthcare system’s services for prostate cancer screening.

### Research hypothesis

1. What is the effect of health belief model-based education on prostate cancer knowledge, health beliefs and preventive health practices among adult and older adult males?
2. Are there age or other independent variables-related differences in relation to perceived knowledge, health beliefs and preventive and screening health practices of prostate cancer screening?

## Materials and methods

### Study design and setting

A one-group pre-test/post-test quasi-experimental study design was used in this study which was conducted at the one-day outpatient clinics affiliated to General Alexandria Main University Hospital. These clinics have a high admission rate and receive patients from all over Alexandria City and the surrounding governorates.

### Target population

The target population were Egyptian men, with the following criteria:

### Inclusion criteria

- Adult men aged between 45 and 75 years old.
- Attended above mentioned setting for either examination or follow-up appointments,
- Able to communicate and accepted to participate in the study.

### Exclusion criteria

- Adult male with current or prior PC diagnosis.
- Severe vision, hearing, or cognitive impairment.
- Previous participation in similar training program.

### Sample size and method of selection

Assuming that the health belief model-based educational program has an effect size = 0.25. The minimum required sample size is 98 individuals using an alpha error of 0.05, study power of 80%, and number of repetitions = 2. The sample size increased to 110 to compensate for the 10% dropout rate. The sample size was calculated using G power version 3.1.9.4 [21]. A convenient sampling method was employed to efficiently select participants from the outpatient clinics, considering their accessibility and availability during the study period. From each clinic eligible participants were recruited into small groups to conduct the intervention sessions until required sample size had been completed.

### Data collection methods and tools

A predesigned structured interview questionnaire was developed to collect the following data (supplementary file 1):

1. **General characteristics** of the study sample including data about age, income, residence, marital status, work status, and level of education, also data about family history of prostate cancer were taken, and physician’s recommendations for prostate cancer screening in regular checkup.
2. **Prostate cancer knowledge questionnaire (PCKQ)**: The questionnaire was adapted from previous studies done by Agho and Lewis (2001) [22] and Weinrich et al. (2007) [23]. PCKQ covers questions related to prevalence, etiology, risk factors, presentations, manifestations, screening practices, and prevention of PC. The questionnaire responses were constructed as true or false or Don’t Know. “Don’t know” responses were treated as incorrect answers. The total score ranged from 26 (maximum) to 0 (minimum). Higher scores indicate higher knowledge.
3. **Prostate cancer screening-health belief model scale (HBM-PCS)**: The Health Belief Model Scale (HBM) has been adapted into the HBM-PCS to assess one’s health beliefs regarding PC screening. Its purpose is to identify perceptions and beliefs that influence an individual’s decision to engage in or avoid preventive services for potential health issues. In 2011, Capik and Gozum [19] developed the HBM-PCS consisting of forty-one items with a five-point Likert scale, ranging from one = completely disagree to five = completely agree. The scale is composed of five sub-scales, including perceived susceptibility (five items), perceived seriousness (four items), health motivations (ten items), perceived barriers (fifteen items), and perceived benefits (seven items). Each sub-scale is scored individually, and a higher score in susceptibility, seriousness, motivation, and benefit, as well as a reversed score in barriers, indicates a positive intervention effect. The HBM-PCS yields a total score by summing the score of sub-scales, with a higher score indicating better outcomes.
4. **Prostate cancer preventive practices questionnaire (PCPPQ)**: Developed by the researcher based on relevant English literature [24–27] to assess respondents’ preventive practices regarding prostate cancer. It consisted of 12 questions and assessed how often the respondents practiced certain preventive behaviors (diet, health-professional help-seeking, and lifestyle modification including smoking cessation/prevention, exercise and physical activity, sleep, stress control, and weight reduction) in day-to-day activities. Participants were given a 4-point Likert scale ranging from never [1] to always [4], to evaluate their responses. A higher total score reflects better preventive practices.
5. **Intention to PC screening question**: Examined the intent to screen (current interest in prostate screening) and screening behavior. Based on Anderson’s study (2013) [28], we used the item ‘Please select a response that best describes your current interest in prostate screening (rectal examination and/or PSA-blood test)’ as the dependent variable. Respondents were asked to choose one from the following options: [1] I have never done them, and I am not planning to do screening, [2] I have never done them, but I am planning to do screening in the upcoming six months, [3] I have done one or both at least once before, and I am intending to have another one on the due date. The responses described if participants had previously undergone a screening test in addition to the assessment of their willingness to or future intention.

### Questionnaire validation

The questionnaire was translated into the Arabic language, and standard translating procedures were followed to ensure precision and consistency. In order to establish inter-rater reliability, two native Arabic translators were engaged to evaluate the translated versions for consistency. A percentage agreement calculation was utilized to compare the translated versions, resulting in an inter-rater agreement of 0.79. To achieve face and content validity, experts from various fields (medical-surgical nursing, gerontological nursing, geriatrician, oncologist, urologist, nursing educator, and public health specialist from Alexandria, Helwan and Cairo Universities), were consulted to evaluate the questionnaire items in terms of simplicity, clarity, relevance, and necessity. Based on their feedback and suggestions, the study tools were amended to ensure content validity. To ensure comprehensibility, intelligibility, and clarity of the questions, a pilot sample of 14 eligible men was selected to provide feedback. Internal consistency was measured using Cronbach’s α values. The calculated Cronbach’s α of the prostate cancer knowledge questionnaire, prostate cancer screening-health belief model scale and prostate cancer preventive practices questionnaire sections were 0.900, 0.691 and 0.790 respectively.

### The intervention program

**Fig. 1:**
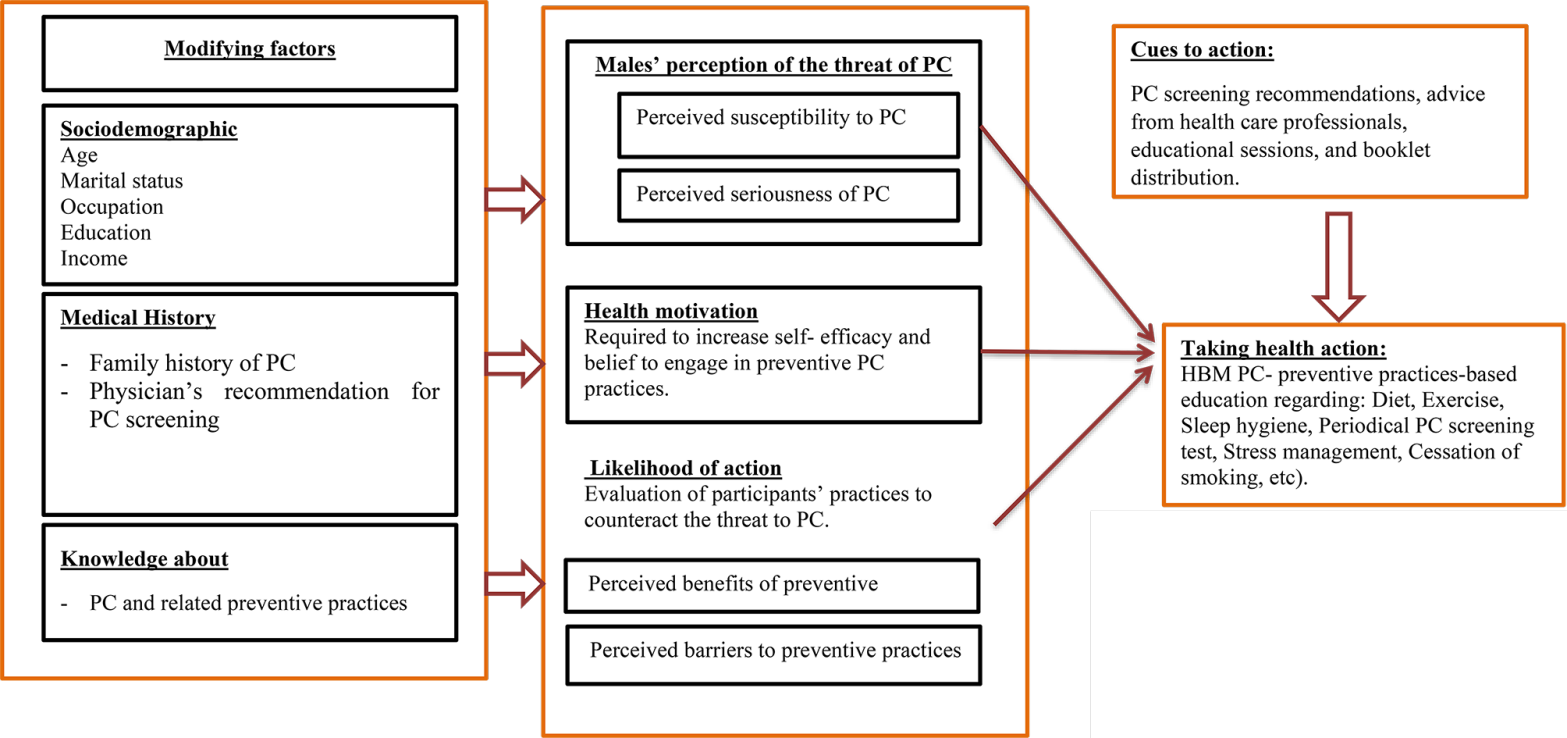
Constructs of the health belief model (HBM) framework applied for adults and older adults’ males related to prostate cancer (PC) preventive educational program

The intervention program comprised of four sessions, each lasting between 30 and 45 min, conducted once a week. (Table 1). The program was developed using the Health Belief Model (HBM) as a framework [29] (Fig. 1). The illiteracy rate in Egypt is quite high, which can pose a challenge to elderly men when it comes to educational materials that are typically in written form. Moreover, visual impairment is commonly associated with ageing, which further exacerbates the issue. To address this concern, a range of educational materials were distributed to study participants. These included pamphlets, as well as links to educational audio formats available on the SoundCloud platform. This was done to cater to the needs of participants who prefer to listen or are unable to read effectively. A booklet containing illustrations about prostate cancer knowledge, prevention and its screening practices were given to the participants, and the information in booklets was explained to the participants during the sessions. The data collection process and program implementation spanned over a duration of 8 months, from March 2022 to February 2023.

**Table 1:** Content of the designed program

**Session (1): “The healthy belief and its importance”**
• The concept of the health belief model
• Objectives of the health belief model
• Health belief model skills
• The necessary steps to develop an action plan.
**Session (2): “Basic information about prostate cancer”**
• An introduction to the prostate and its function
• Definition of prostate cancer
• Causes and risk factors of prostate cancer.
• Clinical presentations of prostate cancer
• Possible methods of treatment if prostate cancer occurs.
**Session (3): “Preventive health practices for prostate cancer”**
• Benefits and importance of prostate cancer screening and preventive practices
• Elements of preventive health practices for prostate cancer • The skills that must be performed to implement the health belief model of PC preventive practices: “Skills to avoid getting ill or decreasing risk” part 1
1. Activities to maintain physical fitness and health:
• The importance of activity and movement to prevent prostate cancer.
• Ways to conserve physical energy.
• Important tips for exercising
• Perform activities of daily living.
• Commitment to the practice of self-care.
2. Commitment to healthy habits:
• Smoking cessation
• The importance of vitamin D and sun exposure
• Having sex
• Rest and sleep
• Doctor consultation
• Proper action in case of abnormal symptoms.
• Increasing knowledge and awareness to correct erroneous negative health ideas and beliefs.
3. Who and When to screen for prostate cancer:
• Introduction to early detection of prostate cancer
• Factors for choosing screening for early detection of prostate cancer.
• The importance of early detection of prostate cancer
• Screening options for early detection of prostate cancer:
• A blood test to measure the level of PSA concentration in the blood.
• Perform a digital rectal examination (palpate the prostate)
• Biopsy and Other examinations
• Maintaining the periodic examination schedule for early detection.
• Proper access to health care institutions.
**Session (4): “Preventive health practices for prostate cancer”** **“Skills to avoid getting ill or decreasing risk” part 2**
4. Proper dieting:
• Proper nutrition and its importance for the prevention of prostate cancer
• Commitment to a healthy diet.
• Elements of proper healthy nutrition
5. Lifestyle and behaviors:
• Social communication with family and friends.
• Linking healthy behaviors to personal goals.
• Learn new skills that make individuals feel good.
• What should be avoided to prevent prostate cancer?
6. Psychological wellbeing
• Be positive about disease prevention.
• Self-confidence and optimism towards the future.
• Looking at life in a positive way.
• Trying to control oneself in the face of psychological problems and pressures.
• Practicing relaxation exercises to reduce anxiety and stress.
• The relationship of anxiety, nervous tension, stress, and psychological state with prostate cancer
• Ways to deal with the stresses of daily life.

N.B., more details of the program could be found in the supplementary data (supplementary file 2)

### Ethical considerations

Approval for the proposed study was granted by the research ethics committee of the esteemed Faculty of Nursing at Alexandria University in Egypt (Decision Date Number: 13/2/2022). All methods were carried out in accordance with the Declaration of Helsinki. In order to ensure ethical standards were met, all participants were provided with written informed consent, and were informed that participation was wholly voluntary, and they could withdraw at any time without any negative consequences. Furthermore, participants were reassured that the information collected would be kept confidential and that no sensitive questions were included, thus ensuring their privacy was protected. Participation was not mandatory, and there was no conflict of interest.

### Statistical analysis

Data collected from the study was meticulously analyzed using the highly regarded IBM SPSS version 25 (SPSS, Inc. Chicago, IL). Continuous variables were presented as mean and standard deviation, while categorical variables were presented as frequencies and percentages. The mean scores of the tested continuous outcome variables were analyzed using a single group design with repeated measurements analysis of variance. To further test for discrepancies in responses across time before and after program completion for dichotomous variables (intention to screen), Cochran’s Q test was employed. All statistical analyses were performed using two-tailed tests, and a p-value less than 0.05 was deemed statistically significant, thus ensuring the utmost accuracy in the findings presented.

## Results

The age of the participants ranged from 45 to 75 years old with nearly equal distribution of all age groups. More than two-thirds of the participants (72.7%) were married, 44.5% were secondary graduates or beyond, and 70.0% were residents of urban areas. Most of the sample (88.2%) were working and 19.1% had an annual income less than 10.000 EGP. Most of the sample (90%) had no family history of prostate cancer (Table 2). No one of the study participants reported that their physician recommends prostate cancer screening during their regular check-up visits.

**Table 2:** General characteristics of the study sample (*n* = 110)

General characteristics	No.	%
**Age**		
▪ 45–49 years	28	25.5
▪ 50–59 years	27	24.5
▪ 60–69 years	29	26.4
▪ 70–75 years	26	23.6
**Marital status**		
▪ Married	80	72.7
▪ Single	7	6.4
▪ Divorced	8	7.3
**Level of education**		
▪ Below secondary level	61	55.5
▪ Secondary level or higher	49	44.5
**Residence**		
▪ Urban	77	70.0
▪ Rural	33	30.0
**Current working status**		
▪ Working	97	88.2
▪ Not working	13	11.8
**Annual income**		
▪ Less than 10.000 EGP	21	19.1
▪ 10.000-less than 20.000 EGP	42	38.2
▪ 20.000-less than 30.000 EGP	26	23.6
▪ 30.000 and more	21	19.1
**Family history of prostate cancer**		
▪ No	99	90.0
▪ Yes	11	10.0

Table 3 shows the mean values of PCKQ, HBM-PCS, and PCPPQ at baseline, after the intervention, and at 3 months follow-up. Participants’ knowledge about prostate cancer screening improved significantly immediately after the program and this positive change was maintained at the follow-up (*p* = 0.000). Furthermore, participants’ perceptions and preventive practices towards prostate cancer screening had changed significantly after program completion and at follow-up (*p* = 0.000).

**Table 3:** Averages scores of the 110 participants with regard to PCKQ, HBM-PCS, and PCPPQ at baseline, post-test and follow up

Variables	Baseline	Post-test	Follow up	F	p-value
	Mean ± SD	Mean ± SD	Mean ± SD		
**PCKQ**	5.84 ± 2.59	15.18 ± 2.47	13.08 ± 2.80	550.24	0.000*
**HBM-PCS**	94.56 ± 16.44	158.55 ± 11.87	138.25 ± 10.35	4281.16	0.000*
**HBM-PCS subscales**					
• Susceptibility	9.88 ± 2.54	19.20 ± 2.17	14.08 ± 2.16	1047.98	0.000*
• Seriousness	7.80 ± 2.11	15.38 ± 2.02	11.41 ± 1.87	809.66	0.000*
• Motivation	24.68 ± 4.55	37.38 ± 4.58	32.64 ± 3.08	739.75	0.000*
• Barriers	31.05 ± 6.64	57.54 ± 5.17	52.47 ± 4.60	2392.24	0.000*
• Benefits	21.15 ± 5.31	29.05 ± 2.67	27.65 ± 2.90	288.21	0.000*
**PCPPQ**	18.53 ± 2.50	30.25 ± 4.94	23.50 ± 5.04	621.48	0.000*
**PCPPQ subscales**					
• Diet	12.24 ± 2.09	19.72 ± 3.43	15.34 ± 3.64	460.97	0.000*
• Change lifestyle	5.11 ± 0.99	8.18 ± 1.53	6.39 ± 1.59	258.12	0.000*
• Health professional help-seeking	1.19 ± 0.42	2.35 ± 0.83	1.77 ± 0.76	132.72	0.000*

F for repeated measure ANOVA, *Significant p value < 0.05

Regarding intention to prostate cancer screening, at baseline most of the participants (78.2%) had never done screening and not planned to do it, however after program completion this percentage changed dramatically (9% immediately after intervention and 23.6% at follow-up, *p* = 0.000). After program completion, many of the participants (92.7%) were planning to screen for prostate cancer in the next six months (*p* = 0.000). No significant change in the intention was noticed in those who did the test once (*p* = 0.069) as shown in Table 4.

**Table 4:** Participants’ intention to prostate cancer screening at baseline, post-test and follow up

Participants’ intention to prostate cancer screening (*n* = 110)	Baseline		Post-test		Follow up		Cochran’s Q	p-value
	No.	%	No.	%	No.	%		
• I’ve never done them, and I am not planning to do it	86	78.2	1	9.0	26	23.6	130.11	0.000*
• I’ve never done them, but I’m planning to do it in the coming six months.	21	19.1	102	92.7	77	70.0	113.42	0.000*
• I do at least once in the past, and I intend to get another	3	2.7	7	6.4	7	6.4	5.33	0.069

*Significant p value < 0.05

Table 5 shows no differences in total knowledge and practice scores among different age groups after the termination of the program. However, the younger age group (45–49 years) showed higher scores in their perception of prostate screening (*p* = 0.001). Higher education and income were significantly associated with higher scores in the three scales (*p* = 0.000 in all scales). Regarding the family history of prostate cancer, no significant differences in knowledge or practice between those who reported having a family history of prostate cancer or not, although a significantly higher perception was found among those who reported having a family history of prostate cancer (*p* = 0.005).

**Table 5:** Averages scores of the 110 participants regarding PCKQ, HBM-PCS, and PCPPQ at post-test in relation to socio-demographic data

Independent variables	PCKQ		F	p-value	HBM-PCS		F	p-value	PCPPQ		F	p-value
	Mean	SD			Mean	SD			Mean	SD		
**Age**			0.597	0.619			6.333	0.001*			2.248	0.087
45–49 years	15.57	2.42			166.14	7.24			32.28	5.18		
50–59 years	14.85	2.24			153.92	12.06			29.70	5.11		
60–69 years	14.89	2.71			157.00	13.08			29.65	4.87		
70–75 years	15.42	2.51			156.88	11.00			29.26	4.17		
**Marital status**			3.005	0.034*			0.889	0.449			1.055	0.372
Married	14.82	2.49			158.77	11.91			30.20	5.40		
Single	14.85	2.19			151.71	11.29			27.57	0.97		
Widowed	16.53	1.84			160.26	11.52			30.86	3.20		
Divorced	16.50	2.56			159.00	12.75			31.87	4.29		
**Level of education**			25.802	0.000*			220.822	0.000*			71.716	0.000*
Below secondary level	14.21	2.18			149.86	6.77			27.45	3.11		
Secondary level or higher	16.38	2.28			169.34	6.90			33.71	4.60		
**Residence**			0.345	0.558			9.099	0.003*			3.691	0.057
Urban	15.27	2.51			160.70	11.93			30.83	5.18		
Rural	14.96	2.39			153.51	10.21			28.87	4.07		
**Current working status**			0.626	0.430			0.388	0.535			1.553	0.215
Working	15.11	2.49			158.80	11.74			30.03	4.80		
Not working	15.69	2.35			156.61	13.05			31.84	5.85		
**Annual income**			5.989	0.000*			13.947	0.000*			10.152	0.000*
Less than 10.000 EGP	13.80	2.04			153.76	10.20			28.19	3.98		
10.000- Less than 20.000 EGP	14.80	2.45			154.07	10.14			28.83	4.22		
20.000-less than 30.000 EGP	15.73	2.14			160.03	10.94			30.57	4.50		
30.000 and more	16.61	2.49			170.42	9.23			34.71	5.07		
**Family history of prostate cancer**			0.411	0.523			8.105	0.005*			3.633	0.059
No	15.13	2.49			157.50	11.85			29.94	4.95		
Yes	15.63	2.33			167.90	7.063			32.90	4.18		

*Significant p value < 0.05

## Discussion

This groundbreaking study on the impact of Health Belief Model (HBM)-based educational intervention on prostate cancer screening in Egypt is a pioneering effort, being the first of its kind. The program proved to be efficacious, with a significant enhancement in participants’ knowledge, perception, and practices concerning prostate cancer prevention and screening. These favorable outcomes were also sustained for a period of three months after the program, implying the potential long-term benefits of such interventions. Furthermore, participants’ intentions for screening positively changed after the completion of the program. Adults were more likely than older adults to report perceived aspects/dimensions for PC screening based on HBM. Those with higher levels of education and annual income exhibited improved knowledge and a positive perception of being engaged in preventive practices than their counterparts.

In the same context, a study conducted in Italy by Maladze et al. (2023) declared that most respondents who received previous information about PC from a physician had moderate knowledge about its screening and management; than those who did not [30]. As per a recent integrative review (2020), the primary barrier preventing men in sub-Saharan Africa from undergoing prostate cancer screening is a lack of knowledge. This is followed by perceptions, attitudes, and beliefs that hinder testing and screening for prostate cancer [31]. In the current study, the reason for the subjects’ low knowledge scores prior to intervention could be attributed to low education level as more than half of the participants did not complete high school (55.5%), also, low income, lacked sufficient PC training programs and the increased focus of health authorities and health care professionals on treatment rather than prevention could be reasons. Absence of mass media orientation campaigns emphasizing PC screening, could predispose adults and older adults to an observable lack of information about the importance of PC screening behaviors and its preventive practices.

In response to this pressing issue, several organizations (Advocacy League, CanSurvive, Egyptian Urology Association, and Astellas Pharma Inc.) have collaboratively launched Prostatecancer.me, the first-ever specialized platform and portal in the Arabic language dedicated to prostate cancer awareness. The platform aims to furnish patients with crucial information on prostate cancer, disease treatment alternatives, possible lifestyle modifications, as well as advice for caregivers. To ensure the platform’s favorable impact on patients and their families, it is imperative to make it more accessible to Arab adults, particularly older adults [32]. 

Applying HBM constructs was useful in predicting the intent to screen, as individuals will take action to prevent, reduce, control, or treat a health problem if they perceive that the action is beneficial and can produce a positive outcome in addition to being more motivated. Based on that premise, it may be useful to frame educational interventions and or prevention strategies in the context of resultant benefits and motivation. Erroneous beliefs undoubtedly influence men’s decision to partake in prostate cancer screening and prevention [33]. Results of our study revealed improvement in the total health belief model average score along all studied assessment periods, within each group in all subscales. Similarly, in a nonexperimental exploratory study conducted by Oliver et al., (2011) a group of 94 male participants aged 40 and above (with a range from 40 to 72 years), residing in rural areas, were examined. The study aimed to investigate the correlation between perceived benefits and perceived barriers with respect to prostate cancer screening decisions and the sources of influence cited by the participants. The results revealed that both benefits and barriers were significantly associated with prostate cancer screening as well as the sources of influence [34]. 

In a similar vein, a descriptive-analytical study was conducted on 263 male employees aged 40 years and older at a medical sciences university in Iran. The primary objective of this study was to understand the preventive behaviors of prostate cancer based on the structures of the health belief model. The study revealed that perceived barriers were high, such as the high cost of choosing a particular diet, lack of exercise and control over body mass index, insufficient knowledge about the place and time for diagnostic tests, a tendency to engage in high-risk behaviors like smoking, and misconceptions about the disease [35]. 

Additionally, the current study illustrated an unsatisfactory practice level in the baseline, which was improved after the intervention, declined again 3 months later but was still significant in comparison with pre-intervention. These findings were supported by Mazloomi, Dehghan and Dehghan (2017) study which suggested that; raising men over 40 years of awareness via HBM education can predispose them to more effective preventive practices; where significant differences in knowledge and practices mean scores were identified between their study participants before and after the intervention, and within both the study and control groups post- intervention; (*p* = 0.000) [36]. 

In the current study, a significant difference was found between different age groups regarding HBM-PCS with the younger age group (45–49 years old) exhibiting better perception, and the lowest score was among those 50–59 years old. This could be explained by the fact that younger age group exhibits a higher level of education, greater access to healthcare resources and health insurance coverage. This group also displays a proactive approach towards their future health through regular screening practices, which has the potential to impact their beliefs and behaviors positively. Lee, Park and Park (2016) declared that age is one of the main factors which significantly needs to be considered in nursing education programs especially in middle-aged men; in order to deliver accurate knowledge about prostate cancer. However, they agreed upon the effectiveness of HBM-based interventions to increase PC screening practices’ sensitivity and reduce barriers in all age groups [37]. 

Incompatible with our finding, Gift and Colleges’ (2020) study on 200 men over 40 years revealed that in the last 2 years, low PC preventive screening practices were observed in their adult participants, while those aged above 60 years had a positive practice (*p* < 0.001); being more knowledgeable about PC screening, maybe because they periodically visit healthcare facilities for other urologic disorders follow up [38]. We also found that higher education and income were positively associated with improvement in knowledge, perception, and practices, this is corroborated by several other studies in Africa [30, 38] Based on an integrative review, it has been suggested that individuals with limited educational attainment may have trouble in comprehending information and may exhibit mistrust toward prostate cancer screening. Additionally, low socio-economic status has been linked to lower uptake of prostate screening and testing [31]. 

HBM educational intervention applied in this study also had a positive impact on participants’ future intention to screen. Jean-Louis & Webb, 2021 study stated that African American men preferred the fecal immunochemical test, stool DNA tests, and Guaiac-based fecal occult blood test because they are noninvasive and of low cost. The use of at-home prostate cancer screening tests enabled routine screening at home, should be disseminated in the community and reinforced by healthcare professionals [39]. 

Despite PC screening guidelines have been advanced by the American Cancer Society (2019) and other organizations (Mason et al., 2022) [40, 41], there is no evidence that Egyptian men participate in routine prostate cancer screening. Within the medical community in Egypt, there exists a lack of consensus and agreement on whether or not to screen for prostate cancer as well as the appropriateness of prostate cancer screening. The current study showed that physicians were not recommending prostate cancer screening on regular follow-ups for all participants, where the majority of them did not perform prostate screening. Kaninjing et al. (2018) affirmed that most of their study participants did not report any screening or recommendation from a healthcare provider for PC annual screening [42]. Furthermore, Naji et al. (2018) stressed upon lack of medical approach for continuous PC screening and periodical follow-up motivation for non-evident PC men aged > 50 years and ≥ 75 years in routine care, in spite of nearly one-third of men in their fifties and up to two third in their seventies have a higher incidence of PC [43]. 

It is important to note that this study does have its limitations, as it lacks a control group. Consequently, it is challenging to make concrete conclusions regarding the efficacy of the intervention. While this study design has been criticized for its inability to determine causality, it is still implemented in various settings to assess educational interventions. Nevertheless, this research could serve as a foundation for future randomized clinical trials aimed at increasing awareness and adoption of prostate cancer screening in rural communes.

## Conclusion

The study findings emphasized the effectiveness of the designed health educational program based on the HBM on PC preventive behaviors, through significantly improving participants’ knowledge level, perceptions, practices, and intentions to PC screening. Thus, HBM-based educational programs are highly recommended for prostate cancer preventive health practices among both adult and older adult males.

### Recommendations

HBM-based health education is warranted to improve knowledge literacy about the PC, dispel misconceptions, and emphasize improving perceived benefits and motivation and identifying health services barriers. The study’s results underscore the significance of providing culturally appropriate healthcare services to the Egyptian population, particularly those at higher risk for prostate cancer, which should be translated into greater access and more effective healthcare. Nurses and physicians must emphasize the health benefits of being examined early, provide motivation, and explain that the person’s masculinity will not be affected. Programs geared toward Egyptian adults and older adults should also concentrate on improving overall health habits, such as quitting smoking, adopting balanced diets, and engaging in physical activity.

### Electronic supplementary material

Below is the link to the electronic supplementary material.

Supplementary Material 1

Supplementary Material 2

## Data Availability

The data that support the findings of this study are available from the corresponding author upon reasonable request.

## Abbreviations

PC: Prostate cancer
HBM: The health belief model
PCKQ: Prostate Cancer Knowledge Questionnaire
HBM-PCS: Prostate Cancer Screening-Health Belief Model Scale
PCPPQ: Prostate Cancer Preventive Practices Questionnaire
IBM: International Business Machines
SPSS: Statistical Package for the Social Sciences

## References

[1] Saifuddin SR, Devlies W, Santaolalla A, Cahill F, George G, Enting D et al. King’s Health Partners’ Prostate Cancer Biobank (KHP PCaBB). BMC cancer. 2017;17(1):784. 10.1186/s12885-017-3773-8. 10.1186/s12885-017-3773-8 PMC5700705 29166865

[2] Wang L, Lu B, He M, Wang Y, Wang Z, Du L (2022). Prostate Cancer incidence and mortality: global status and temporal trends in 89 countries from 2000 to 2019. Front Public Health.

[3] Sung H, Ferlay J, Siegel RL, Laversanne M, Soerjomataram I, Jemal A et al. Global Cancer Statistics 2020: GLOBOCAN Estimates of Incidence and Mortality Worldwide for 36 Cancers in 185 Countries. CA: a cancer journal for clinicians. 2021;71(3):209– 49. 10.3322/caac.21660. 10.3322/caac.21660 33538338

[4] Fitzmaurice C, Allen C, Barber RM, Barregard L, Bhutta ZA, Brenner H et al. Global, Regional, and National Cancer Incidence, Mortality, Years of Life Lost, Years Lived With Disability, and Disability-Adjusted Life-years for 32 Cancer Groups, 1990 to 2015: A Systematic Analysis for the Global Burden of Disease Study. JAMA oncology. 2017;3(4):524– 48. 10.1001/jamaoncol.2016.5688. 10.1001/jamaoncol.2016.5688 PMC6103527 27918777

[5] World Health Rankings. Available at https://www.worldlifeexpectancy.com/egypt-prostate-cancer, 2018.

[6] World Health Organization/International Agency for Research on Cancer: Egypt. December 2020. Available at https://gco.iarc.fr/today/data/factsheets/populations/818-egypt-fact-sheets.pdf. Accessed March 5, 2021.

[7] Rebbeck TR, Devesa SS, Chang BL, Bunker CH, Cheng I, Cooney K, et al. Global patterns of prostate cancer incidence, aggressiveness, and mortality in men of African descent. Prostate cancer. 2013;2013(560857). 10.1155/2013/560857. 10.1155/2013/560857 PMC3583061 23476788

[8] Cancer Research UK. Cancer Statistics for the UK 2015 [cited 2015 27th July].

[9] Lortet-Tieulent J, Georges D, Bray F, Vaccarella S. Profiling global cancer incidence and mortality by socioeconomic development. International journal of cancer. 2020;147(11):3029– 36. 10.1002/ijc.33114. 10.1002/ijc.33114 32449164

[10] Baratedi WM, Tshiamo WB, Mogobe KD, McFarland DM (2020). Barriers to prostate Cancer screening by men in Sub-saharan Africa: an Integrated Review. J Nurs Scholarsh.

[11] Persaud H, Yuan J, Afable A, Bruno DM. Barriers to Prostate Cancer Screening Among Indo-Guyanese. Journal of Community Health. 2021;46(3):591– 6. 10.1007/s10900-020-00926-5. 10.1007/s10900-020-00926-5 32960396

[12] ASAm Emezayen, Fouda LM, Essa HAEG, El mezayen SES. The Effect of Educational Program on Knowledge and Commitment of Male Employees at Tanta University Regarding Prostate Cancer Screening %J Tanta Scientific Nursing Journal. 2022;25(2):68-81. 10.21608/tsnj.2022.241904.

[13] Kanungo S, Bhowmik K, Mahapatra T, Mahapatra S, Bhadra UK, Sarkar K. Perceived morbidity, healthcare-seeking behavior and their determinants in a poor-resource setting: observation from India. PloS one. 2015;10(5):e0125865. 10.1371/journal.pone.0125865. 10.1371/journal.pone.0125865 PMC4428703 25965382

[14] Morrison BF, Aiken WD, Mayhew R, Gordon Y, Odedina FT. Prostate Cancer Knowledge, Prevention, and Screening Behaviors in Jamaican Men. Journal of cancer education: the official journal of the American Association for Cancer Education. 2017;32(2):352– 6. 10.1007/s13187-016-0991-8. 10.1007/s13187-016-0991-8 PMC5553046 26842816

[15] American Cancer Society. Cancer Facts & Fig. 2021. Atlanta, Ga: American Cancer Society; 2021.

[16] Egyptian Ministry of Health and Population, World Health Organization.: Egypt Multisectoral Action Plan for Noncommunicable Diseases: Prevention and Control, 2018–2022. Available at http://158.232.12.119/ncds/governance/policies/Egypt-NCD-MAP-2018-2022.pdf. Accessed January 19, 2021.

[17] World Health Organization. Regional Office for the Western P. Sustainable development goals (SDGs): Goal 3. Target 3.4: By 2030, By 2030, reduce by one third premature mortality from non-communicable diseases through prevention and treatment and promote mental health and well being [poster]. Manila: WHO Regional Office for the Western Pacific; 2016, 2016.

[18] Riekert KA, Ockene JK, Pbert L. The handbook of health behavior change. Springer Publishing Company; 2013.

[19] Çapık C, Gözüm S (2011). Development and Validation of Health beliefs Model Scale for prostate Cancer screenings (HBM-PCS): evidence from exploratory and confirmatory factor analyses. Int J Cancer Biomedical Res.

[20] Friedman DB, Thomas TL, Owens OL, Hébert JR (2012). It takes two to talk about prostate Cancer: a qualitative Assessment of African American men’s and women’s Cancer Communication practices and recommendations. J Cancer Education: Official J Am Association Cancer Educ.

[21] Berger J, Bayarri M, Pericchi LJER (2014). Effective Sample size.

[22] Agho AO, Lewis MA (2001). Correlates of actual and perceived knowledge of prostate Cancer among African americans. Cancer Nurs.

[23] Weinrich SP, Seger R, Curtsinger T, Pumphrey G, NeSmith EG, Weinrich MC (2007). Impact of Pretest on Posttest Knowledge scores with a Solomon Four Research Design. Res Theory Nurs Pract.

[24] National Cancer Institute. Physician Data Query (PDQ). Prostate Cancer Prevention. 2019. Accessed at https://www.cancer.gov/types/prostate/hp/prostate-prevention-pdq on March 28, 2019.

[25] Rock CL, Thomson C, Gansler T, Gapstur SM, McCullough ML, Patel AV, Andrews KS, Bandera EV, Spees CK, Robien K, Hartman S, Sullivan K, Grant BL, Hamilton KK, Kushi LH, Caan BJ, Kibbe D, Black JD, Wiedt TL, McMahon C, Sloan K, Doyle C (2020). American Cancer Society guideline for diet and physical activity for cancer prevention. CA Cancer J Clin.

[26] Brookman-May SD, Campi R, Henriquez JD, Klatte T, Langenhuijsen JF, Brausi M et al. Latest evidence on the impact of smoking, sports, and sexual activity as modifiable lifestyle risk factors for prostate cancer incidence, recurrence, and progression: a systematic review of the literature by the European Association of Urology Section of Oncological Urology (ESOU). 2019;5(5):756–87. 10.1016/j.euf.2018.02.007 29576530

[27] Vasconcelos A, Santos T, Ravasco P, Neves PM (2019). Dairy products: is there an impact on Promotion of prostate Cancer? A review of the literature. Front Nutr.

[28] Anderson MM. Testing the health belief model using prostate cancer screening intention: comparing four statistical approaches applied to data from the 2008-09 Nashville Men’s Preventive Health Survey. Journal of Men’s Health; 2013.

[29] Zare M, Ghodsbin F, Jahanbin I, Ariafar A, Keshavarzi S, Izadi T (2016). The Effect of Health Belief Model-based education on knowledge and prostate Cancer screening behaviors: a Randomized Controlled Trial. J Interventional Cancer Epidemiol Prev.

[30] Maladze N, Maphula A, Maluleke M, Makhado L (2023). Knowledge and attitudes towards prostate Cancer and screening among males in Limpopo Province, South Africa. Int J Environ Res Public Health.

[31] Baratedi WM, Tshiamo WB, Mogobe KD, McFarland DM (2020). Barriers to prostate cancer screening by men in Sub-saharan Africa: an integrated review. J Nurs Scholarsh.

[32] ProstateCancer.me Launched to Address Issues Identified in the First Prostate Cancer Patient Survey. Homepage. Retrieved from https://www.prnewswire.com/ae/news-releases/prostatecancerme-launched-to-address-issues-identified-in-the-first-prostate-cancer-patient-survey-300929545.html.

[33] Bilgili N, Kitiş Y. Prostate Cancer Screening and Health beliefs: a Turkish study of male adults. Eur J Med Health Sci. 2019;41(2).

[34] Oliver JS, Grindel CG, DeCoster J, Ford CD, Martin MY. Benefits, barriers, sources of influence, and prostate cancer screening among rural men. Public Health Nurs. 2011 Nov-Dec;28(6):515–22. 10.1111/j.1525-1446.2011.00956.x 22092461

[35] Najimeh Beygi Z, Alizadeh Z, Fereidouni T, Mokhlesabadifarahani NNN, Abadi S, Soudagar, Afsaneh Ghasemi. Evaluation of the Effect of Health Belief Model Based Training on Health Performance of Male Staff in Fasa University of Medical Science in the field of prostate Cancer. J Complement Med Res. 12(3), 158–64. 10.5455/jcmr.2021.12.03.21.

[36] Dehghan HJT (2017). Effect of education on preventive treatment of prostate cancer in men over 40 years of Yazd. Health Belief Model.

[37] Lee E, Park Y, Park JJOJN. Knowledge, health beliefs and screening status of prostate cancer among middle-aged and elderly men. 2016;6(9):672–87.

[38] Gift S, Nancy K, Victor MJAJU (2020). Assessment of knowledge, practice and attitude towards prostate cancer screening among male patients aged 40 years and above at Kitwe Teaching Hospital. Zambia.

[39] Jean-Louis A, Webb FJ (2021). Knowledge, preferences and willingness to use at-home prostate and colorectal cancer screening tests in African American and Haitian men. Ecancermedicalscience.

[40] American Cancer Society. (2019). Causes, risk factors, and prevention. https://www.cancer.org/cancer/prostate-cancer/causes-risks-prevention/risk.

[41] Mason RJ, Marzouk K, Finelli A, Saad F, So AI, Violette PD, Breau RH, Rendon RA (2022). UPDATE– 2022 Canadian Urological Association recommendations on prostate cancer screening and early diagnosis endorsement of the 2021 Cancer Care Ontario guidelines on prostate multiparametric magnetic resonance imaging. Can Urol Assoc J.

[42] Kaninjing E, Lopez I, Nguyen J, Odedina F, Young ME (2018). Prostate Cancer screening perception, beliefs, and practices among men in Bamenda, Cameroon. Am J Mens Health.

[43] Naji L, Randhawa H, Sohani Z, Dennis B, Lautenbach D, Kavanagh O, Bawor M, Banfield L, Profetto J (2018). Digital rectal examination for prostate Cancer screening in primary care: a systematic review and Meta-analysis. Ann Fam Med.

