# Supplementary material for: Effect of health belief model-based educational intervention on prostate cancer prevention; knowledge, practices, and intentions
Source: BMC Cancer. 2024 Mar 4;24:289. doi: 10.1186/s12885-024-12044-9 (PMC10913411; doi:10.1186/s12885-024-12044-9)
Supplement: Supplementary file 1 — Supplementary Material 1 [file 12885_2024_12044_MOESM1_ESM.docx]

**Constructs of the HBM applied for providing prostate cancer preventive health practices for adults and older adults patients in outpatient clinics**

| **The overall goal of the program** | The program aims to assess the impact of health belief model-based learning on preventive health practices for prostate cancer | | | |
| --- | --- | --- | --- | --- |
| **Target group** | Men from 45 to 75 years old | | | |
| **Number of sessions** | (4) individual sessions, at the rate of (1) session per week, at the rate of two hours for the total sessions, and they are given face to face with the follow of the precautionary measures | | | |
| **Session (1):**  **Introductory session** | Session Title: **“The healthy belief and its importance”**  Session duration: **30 minutes**  Session Content:   - The concept of the health belief model - Objectives of the health belief model - Health belief model skills - The necessary steps to develop an action plan. | | | |
| **Health Belief Model Constructs** | **Definition** | **Objectives** | **Session**  **content** | **Application to prostate cancer education** |
| Perceived susceptibility | One’s opinion of his chances of getting a  Condition  (Perception of risk of prostate cancer) | Gain information about possible risk factors of prostate cancer, and how to develop an action plan to apply skills to initiate health beliefs. | **Session (2):**  Session Title: **“Basic information about prostate cancer”**  Session duration: **30 minutes**  Session Content:   - An introduction to the prostate and its function - Definition of prostate cancer - Causes / risk factors of prostate cancer. - Symptoms of prostate cancer - Possible methods of treatment if prostate cancer occurs. | - Personalize risk of prostate cancer based on person’s family history or behaviors. - Raise perceived susceptibility, awareness of prostate cancer prevention, provide information about risk factors and what symptoms occur if prostate cancer develops (knowledge of prostate cancer risk-factors by which prostate risk can be high including age, race, family history, lifestyle, and body mass index). |
| Perceived seriousness | One’s belief that his condition has serious consequences (e.g., death).  (One’s opinion of how serious a condition and its sequelae are) | Improve knowledge regarding consequences or complications affecting patient everyday livings if prostate cancer presents | - Complications of prostate cancer and its treatments | - Specify consequences of the risk of prostate cancer and complications that results from prostate cancer if develops. - Perceive of seriousness of risk- that prostate cancer may lead to death. |
| Perceived  barriers | One’s opinion of the tangible and psychological  costs of the advised action | Anticipate benefits from trying to prevent prostate cancer to self-outweigh anticipated costs of doing so | - Barriers to engage in prostate cancer preventive and screening practices i.e., lack of access to health care, socioeconomic status or cost, transportation, inadequate knowledge, fear and worry, lack desire, belief of inaccuracy of tests, patient-provider communication, distrust of the medical profession, and aversion to digital rectal exam have been identified as possible barriers to prostate cancer screening. - Different prostate cancer prevention strategies (overcome barriers) | Identify and reduce barriers to engaging in prostate cancer risk reduction and preventive strategies through reassurance, incentives, and support from staff and family |
| Perceived  benefits | One’s opinion of the efficacy of the advised action to reduce risk or seriousness of impact | Belief that strategies intended to prevent prostate cancer will reduce risk of prostate cancer. | **Session (3):**  Session Title: **“Preventive health practices for prostate cancer”**  Session duration: **30 minutes**  Session Content:   - Benefits and importance of prostate cancer screening and preventive practices - Elements of preventive health practices for prostate cancer - **The skills that must be performed to implement the health belief model of PC preventive practices:**   (Skills to avoid getting ill or decreasing risk):  1- Skills of follow a sporting activity to maintain physical fitness and health:   - The importance of activity and movement to prevent prostate cancer. - Ways to conserve physical energy. - Important tips for exercising - Perform activities of daily living. - Commitment to the practice of self-care.   2- Skills of Commitment to healthy habits:   - smoking cessation - The importance of vitamin D and sun exposure - Having sex - Rest and sleep - Doctor consultation - Proper action in case of abnormal symptoms. - Increasing knowledge and awareness to correct erroneous negative health ideas and beliefs.   3- Skills of Who and When to screen for prostate cancer:   - Introduction to early detection of prostate cancer - Factors for choosing screening for early detection of prostate cancer. - The importance of early detection of prostate cancer   **Screening options for early detection of prostate cancer:**   - A blood test to measure the level of PSA concentration in the blood. - Perform a digital rectal examination (palpate the prostate) - Biopsy and Other examinations - Maintaining the periodic examination schedule for early detection. - Proper access to health care institutions. | Define action to take to reduce prostate cancer risk, clarify the  positive effects on reassurance, early detection, being curable, decrease spreading, safety, and lower the chance of death to be expected.  Clarify the importance of adopting a healthy lifestyle, being active, performing self – care practices, and maintaining physical activity.  Illustrate the benefits of avoid high-risk behaviors and maintain sex, rest and sleep, and being careful of abnormal manifestations to correct negative beliefs and initiate health behaviors. |
| health motivation | One’s state of intent that results in behaviors to participate in strategies to activate readiness to change.  i.e., engage in prostate cancer strategies, motivation to take action | - Initiate intent to perform personal action plan by participation in prostate cancer prevention actions.  - Belief in own ability to attempt prostate cancer prevention strategies | **Session (4):**  Session Title: **“Preventive health practices for prostate cancer”**  Session duration: **30 minutes**  Session Content:  4- Skills of proper dieting:   - Proper nutrition and its importance for the prevention of prostate cancer - Commitment to a healthy diet. - Elements of proper healthy nutrition   5- Skills related to lifestyle and behaviors:   - Social communication with family and friends. - Linking healthy behaviors to personal goals. - Learn new skills that make individuals feel good. - What should be avoided to prevent prostate cancer.   6- Skills of dealing with the psychological condition:   - Be positive about disease prevention. - Self-confidence and optimism towards the future. - Looking at life in a positive way. - Trying to control oneself in the face of psychological problems and pressures. - Practicing relaxation exercises to reduce anxiety and stress. - The relationship of anxiety, nervous tension, stress and psychological state with prostate cancer - Ways to deal with the stresses of daily life. - The relationship of anxiety, nervous tension, pressures, and the psychological state to prostate cancer. | Provide how-to information about the strategies, promote awareness (such as provide booklet, provide a man considering prostate cancer screening the opportunity to engage in a decision-making and support process that is shared with a healthcare provider, introduction of choices and available options, emphasize confidence and trust-building relationships). Training and support for performing prostate cancer prevention behaviors, with staff, educator, family provide positive feedback on actions taken. |
