# Supplementary material for: Effect of health belief model-based educational intervention on prostate cancer prevention; knowledge, practices, and intentions
Source: BMC Cancer. 2024 Mar 4;24:289. doi: 10.1186/s12885-024-12044-9 (PMC10913411; doi:10.1186/s12885-024-12044-9)
Supplement: Supplementary file 2 — Supplementary Material 2 [file 12885_2024_12044_MOESM2_ESM.docx]

**Effect of Health Belief Model-Based Educational Intervention on Prostate Cancer Prevention; Knowledge, Practices, and Intentions**

**Dears Adult / Older Adult Participants**

Thank you for giving consideration to participating in this research.

My name is Marwa Ibrahim Mahfouz, Assistant Professor, at the Faculty of Nursing, Alexandria University, Egypt.

All data collected through research tools are kept in strict confidentiality and are only viewed by researchers.

You have the right to agree to participate in the research or to withdraw at any time without giving reasons. Please read the following sentences carefully and sign them before starting to answer.

All data for my participation in the research has been explained:

- I was allowed to ask the researchers any questions I felt were important.
- I know that I can refuse to participate or withdraw at any time without giving reasons.
- All my personal information is confidential, but the data may be shared to serve the research and educational goals as the researchers see.

The information obtained from this research will be used for publication in journals and/or presented at conferences.

This research aims to determine the effect of health belief model-based teaching on preventive health practices regarding prostate cancer among males. All participants need to complete a total of 88-item interviewer-administered pretest questionnaire, which is evaluated and considered culturally appropriate and easy to understand. The topics of the questionnaire are related to socio-demographic information, specific questions are about knowledge, preventive behaviors regarding prostate, and screenings (47 items), in addition to HBM-PCS (41 items). The post-test will be given to the participants immediately after and three months following the educational intervention. The questionnaire will be assigned a numerical code to ensure confidentiality.

| **Name:** | **Date:** |
| --- | --- |
| **Mobile Phone:** | **Signature:** |

**The first tool: Respondents Socio-Economic and Demographic Characteristics**

**1) Age:** ………….

(a) 40-49 years old ( ), (b) 50-59 years old ( ),

(c) 60-69 years Old ( ), (d) 70 to 75 years old ( ).

**2) Annual Income level ranges:**…………..

(a) less than 10.000LE ( ) (b)10.000- less than 20.000 L.E ( )

(b) 20.000-less than 30.000 LE ( ) (d) 30.000 and more LE ( )

**3) Residence:** (a) Urban ( ) (b) Rural ( )

**4) Marital Status:** (a) Married ( ) (b) Single ( ) (c) Widowed ( )

(d) Separated ( ) (e) Divorced ( )

**5) Current Occupation:** (a) Retired ( ) (b) Still work ( ) (c) Doesn’t work ( )

**6) Level of Education:**

1) Illiterate/never attend schools ( ) 2) Primary ( ) 3) Secondary/Diplomat ( ) 4) University, Diploma, master or doctoral degree ( )

**7) When you have a regular check-up or clinical assessment, does your doctor recommend prostate exams?**

1) Yes ( ) 2) No ( )

**8) Family history:**

Do you have a family history of prostate cancer? 1) Yes ( ) 2) No ( )

**9) The second tool: Respondent’s knowledge of Prostate screening testing (With answer keys)**

| **Statements** | **True** | **False** | **I don’t know** |
| --- | --- | --- | --- |
| **1) Prostate cancer is the second most common cancer diagnosis among men** | **True** | **False** | **I don’t know** |
| **2) The cause of prostate cancer is unknown** | **True** | **False** | **I don’t know** |
| **3) Younger men are more likely to develop prostate cancer than older men** | **True** | **False** | **I don’t know** |
| **4) Prostate cancer only affects men over the age of 65** | **True** | **False** | **I don’t know** |
| **5) Men who have first-degree relatives with prostate cancer are more likely to develop it.** | **True** | **False** | **I don’t know** |
| **6) A man can develop prostate cancer without showing any symptoms or pain** | **True** | **False** | **I don’t know** |
| **7) Removing a sample of cells from your body is the only way to know if you have prostate cancer.** | **True** | **False** | **I don’t know** |
| **8) A digital rectal exam can be used to detect prostate cancer** | **True** | **False** | **I don’t know** |
| **9) The prostate-specific antigen (PSA) blood test can be used to detect prostate cancer.** | **True** | **False** | **I don’t know** |
| **10) Prostate cancer can be prevented** | **True** | **False** | **I don’t know** |
| **11) A high-fat diet increases your chances of developing prostate cancer.** | **True** | **False** | **I don’t know** |
| **12) Regular physical activity and maintaining a healthy weight can reduce the risk of prostate cancer.** | **True** | **False** | **I don’t know** |
| **13) The earlier prostate cancer is detected, the better the chances of effective treatment.** | **True** | **False** | **I don’t know** |
| **14) Psychological stress increases the chances of developing prostate cancer** | **True** | **False** | **I don’t know** |
| **15) Most 80-year-old men do not need to be screened for prostate cancer.** | **True** | **False** | **I don’t know** |
| **16) A healthcare provider should advise men 45 years of age or older to have a regular prostate exam** | **True** | **False** | **I don’t know** |
| **17) Tests for prostate cancer are only required when a person has symptoms or problems** | **True** | **False** | **I don’t know** |
| **18) An annual rectal exam or an annual blood test for prostate cancer is recommended starting at age 60** | **True** | **False** | **I don’t know** |
| **19) Men can get cancer and have a normal PSA blood test.** | **True** | **False** | **I don’t know** |
| **20) Prostate cancer may grow slowly in some men** | **True** | **False** | **I don’t know** |
| **21) The only way to detect prostate cancer early, is through regular screening.** | **True** | **False** | **I don’t know** |
| **Which of the following could be a sign of prostate cancer? (Please Check all that apply)** | | | |
| **22) Difficulty in the flow of urine or inability to urinate ( )** | **True** | **False** | **I don’t know** |
| **23) Blood in the urine or semen ( )** | **True** | **False** | **I don’t know** |
| **24) Erectile dysfunction ( )** | **True** | **False** | **I don’t know** |
| **25) Unexpected bladder infections ( )** | **True** | **False** | **I don’t know** |
| **26) Pain when urinating or ejaculating ( )** | **True** | **False** | **I don’t know** |

**10) The third tool: Health Belief Model – Prostate Cancer Scale**

**Subscales, n of items, Minimum and Maximum scores of HBM-PCS**

| **Items** | **Subscale** | **n of Items** | **Min. Point** | **Max. Point** |
| --- | --- | --- | --- | --- |
| Items 1-5 | **Susceptibility** | 5 | 5 | 25 |
| Items 6-9 | **Seriousness** | 4 | 4 | 20 |
| Items 10-19 | **Motivation** | 10 | 10 | 50 |
| Items 20-34 | **Barriers** | 15 | 15 | 75 |
| Items 35-41 | **Benefits** | 7 | 7 | 35 |

| **SUSCEPTIBILITY** | **Strongly**  **Dis-**  **agree**  **(1)** | **Dis-agree**  **(2)** | **Neither Agree**  **nor Disagree**  **(3)** | **Agree**  **(4)** | **Strongly**  **Agree**  **(5)** |
| --- | --- | --- | --- | --- | --- |
| 1. I have a high probability of having prostate cancer. |  |  |  |  |  |
| 1. I have a high probability of having prostate cancer in the next few years. |  |  |  |  |  |
| 1. I have a feeling that I will have prostate cancer at some time in my life. |  |  |  |  |  |
| 1. I fear that I may die because of prostate cancer. |  |  |  |  |  |
| 1. I have a high probability of having prostate cancer when compared to other men of my age. |  |  |  |  |  |
| **SERIOUSNESS** | **Strongly**  **Dis-**  **agree**  **(1)** | **Dis-agree**  **(2)** | **Neither Agree**  **nor Disagree**  **(3)** | **Agree**  **(4)** | **Strongly**  **Agree**  **(5)** |
| 1. It frightens me to think of prostate cancer. |  |  |  |  |  |
| 1. I will experience several problems for a long time if I have prostate cancer. |  |  |  |  |  |
| 1. Prostate cancer will have a negative effect on my relationship with my wife or partner. |  |  |  |  |  |
| 1. My whole life will change in a negative way if I have prostate cancer. |  |  |  |  |  |
| **MOTIVATION** |  |  |  |  |  |
| 1. I follow new information and developments in order to improve my health. |  |  |  |  |  |
| 1. I believe that it is important to perform activities to improve my health. |  |  |  |  |  |
| 1. I keep a balanced diet. |  |  |  |  |  |
| 1. I do sports at least 3 times a week. |  |  |  |  |  |
| 1. I have my medical check-ups regularly even if I am not sick. |  |  |  |  |  |
| 1. It is easy for me to plan to participate in prostate cancer screenings (rectal examination and blood test performed by taking blood sample, PSA measurement). |  |  |  |  |  |
| 1. Participating in prostate cancer screenings will contribute to my health. |  |  |  |  |  |
| 1. I want to have blood test [PSA] for prostate cancer in the next 6 months. |  |  |  |  |  |
| 1. I want to have prostate examination in the next 6 months. |  |  |  |  |  |
| 1. If I have prostate cancer; I want to know it as soon as possible. |  |  |  |  |  |
| **BARRIERS** | **Strongly**  **Dis-**  **agree**  **(1)** | **Dis-agree**  **(2)** | **Neither Agree**  **nor Disagree**  **(3)** | **Agree**  **(4)** | **Strongly**  **Agree**  **(5)** |
| 1. I fear prostate cancer screenings because I do not know how it is performed. |  |  |  |  |  |
| 1. I do not know where and how to go for prostate cancer screenings. |  |  |  |  |  |
| 1. It takes a lot of time to participate in prostate cancer screenings. |  |  |  |  |  |
| 1. I forget to participate in prostate cancer screenings. |  |  |  |  |  |
| 1. I have more important problems than participating in prostate cancer screenings. |  |  |  |  |  |
| 1. I do not know whether the health insurance covers prostate cancer screenings. |  |  |  |  |  |
| 1. I do not know which specialist to see for prostate cancer screenings. |  |  |  |  |  |
| 1. I fear participating in prostate cancer screenings because I feel that something is wrong. |  |  |  |  |  |
| 1. If I am diagnosed with prostate cancer after prostate cancer screenings; there will be nothing to do for its treatment. |  |  |  |  |  |
| 1. I do not need to participate in prostate cancer screenings, since I am not experiencing any problems. |  |  |  |  |  |
| 1. I fear that the results of prostate cancer screening will be bad. |  |  |  |  |  |
| 1. Prostate examination is very unsettling. |  |  |  |  |  |
| 1. Prostate examination is very painful. |  |  |  |  |  |
| 1. Doctors who perform the prostate examination treat patients impolite. |  |  |  |  |  |
| 1. Sexual ability declines after prostate cancer treatment. |  |  |  |  |  |
| **BENEFITS** | **Strongly**  **Dis-**  **agree**  **(1)** | **Dis-agree**  **(2)** | **Neither Agree**  **nor Disagree**  **(3)** | **Agree**  **(4)** | **Strongly**  **Agree**  **(5)** |
| 1. I will be doing something good for myself if I participate in prostate cancer screenings. |  |  |  |  |  |
| 1. If I participate in prostate cancer screenings and if I do not receive any diagnosis, I won’t have to worry about prostate cancer. |  |  |  |  |  |
| 1. Participating in prostate cancer screenings will help an early diagnosis of cancer. |  |  |  |  |  |
| 1. If prostate cancer is diagnosed early and if it is treated successfully, I will have a chance to live a long life. |  |  |  |  |  |
| 1. If prostate cancer screenings do not reveal any negative results; I will know that I am healthy. |  |  |  |  |  |
| 1. If prostate cancer is diagnosed early; the growth of cancer may be prevented by treatment. |  |  |  |  |  |
| 1. If I participate in prostate cancer screenings; I will know the truth about my health condition. |  |  |  |  |  |

**11) The fourth tool: A questionnaire of preventive practices for prostate cancer for adults and older adults:**

| **Statements** | **1) never** | **2) sometimes** | **3) often** | **4) routinely** |
| --- | --- | --- | --- | --- |
| 1) I choose a diet that is low in fat, sugar, processed foods, and cholesterol. | **1** | **2** | **3** | **4** |
| 2) I don't smoke/I plan to stop smoking because I know it's a risk factor for prostate cancer | **1** | **2** | **3** | **4** |
| 3) I have restricted or reduced my consumption of dairy products and red meat | **1** | **2** | **3** | **4** |
| 4) I try to include vegetables in my diet on a daily basis | **1** | **2** | **3** | **4** |
| 5) I discuss my health concerns with health professionals | **1** | **2** | **3** | **4** |
| 6) I get an adequate amount of sleep during the night and avoid staying up late | **1** | **2** | **3** | **4** |
| 7) I avoid exposure to any psychological pressure | **1** | **2** | **3** | **4** |
| 8) I exercise daily with exposure to sunlight and pay attention to activity and movement for a period of not less than 30 minutes. | **1** | **2** | **3** | **4** |
| 9) I avoid charred meat from frying or grilling at high temperatures | **1** | **2** | **3** | **4** |
| 10) I add green tea and soybeans to my food. | **1** | **2** | **3** | **4** |
| 11) I maintain an appropriate and healthy weight and avoid obesity | **1** | **2** | **3** | **4** |
| 12) Focus on healthy fats such as omega-3 fatty acids (nuts and seeds) and fish (sardines, tuna, mackerel, salmon). | **1** | **2** | **3** | **4** |

**12) The fifth tool: The intention to screening for prostate cancer:**

**Please select a response that best describes your current interest in prostate screening (rectal examination and/or PSA-blood test):**

1. I have never done them, and I am not planning to do screening.
2. I have never done them, but I am planning to do screening in the upcoming six months.
3. I have done one or both at least once before, and I am intending to have another one on the due date.

**Thank you for your interest and participation.**
